# Vitamin D (25(OH)D) in patients with chronic kidney disease stages 2-5

**Published:** 2016-09-30

**Authors:** César Augusto Restrepo Valencia, José Vicente Aguirre Arango

**Affiliations:** 1 Universidad de Caldas, Manizales, Colombia; 2 Universidad de Manizales, Manizales, Colombia

**Keywords:** Kidney failure, chronic, vitamin D deficiency, hyperparathyroidism, calcitriol, ergocalciferols, skin pigmentation, renal insufficiency

## Abstract

**Objective:**

To establish the impact the chronic kidney disease stage has in the native vitamin D levels in patients not undergoing dialysis treatment.

**Methods:**

A study performed in Manizales, Colombia, a city located 2,200 meters above sea level, without important stational variations. Patients with 18 years of age or more, with chronic kidney disease stages 2 to 5 and not undergoing dialysis treatment were recruited for this study. Demographic and anthropometric variations were evaluated as well as solar exposure, CKD etiology and laboratory variables related to bone and mineral diseases. For each CKD clinical stage, correlations were evaluated for vitamin D levels, laboratory results for bone and mineral diseases, solar exposure and ethnicity.

**Results:**

Three hundred thirty-three patients were evaluated with a median age of 71 years, most of them mestizo (71%), 173 were women. The main CKD etiology was hypertensive nephropathy (32.2%). 21.1% of patients had normal vitamin D levels, 70.1% were within insufficient range and 8.8% were in deficit. A negative correlation was found between the levels of vitamin 25 (OH) D and the values for: creatinine, phosphorous, calcium x phosphorous product, PTH, 24 hours urine protein and BMI. A positive relationship was found for calcium and albumin. Positive significant statistical correlation was found for vitamin 25(OH) D levels and solar exposure for stages 3b and 4 of CKD.

**Conclusions:**

It is common to find low levels of vitamin 25(OH) D in patients with CKD; these can contribute to the appearance of secondary hyperparathyroidism.

## Abstract (es)

**Objetivo:**

Establecer el impacto del estadio clínico en los niveles de vitamina D nativa en pacientes con enfermedad renal crónica (ERC) sin diálisis.

**Métodos:**

Estudio realizado en Manizales, Colombia, una ciudad tropical ubicada a 2,200 metros de altura sobre el nivel del mar, sin variaciones estacionales importantes a lo largo del año. Se incluyeron pacientes mayores de 18 años, con enfermedad renal crónica estadio 2 a 5 sin tratamiento dialítico. En ellos se evaluaron variables demográficas, antropométricas, grado de exposición solar, etiología de la enfermedad, y variables de laboratorio relacionadas con desórdenes óseos y minerales. Para cada estadío clínico se evaluó la correlación entre los niveles de vitamina D y los resultados de las pruebas de laboratorio relacionadas con desordenes óseos y minerales, exposición solar y etnia.

**Resultados:**

Se evaluaron 331 pacientes, con una edad media de 71 años, la mayoría mestizos (71%), 173 mujeres. La principal etiología de ERC fue nefropatía hipertensiva (33.2%). El 21.1% de los pacientes tenían niveles normales de vitamina D, fueron insuficientes en 70.1% y 8.8% en déficit. Se detectó correlación negativa, entre los niveles de vitamina 25(OH)D y los valores de creatinina, fósforo, producto calcio x fósforo, PTH, proteínas en orina de 24 horas e IMC. Correlación positiva para el calcio y la albumina. Se encontró significancia estadística positiva entre los niveles de vitamina 25(OH)D y la exposición solar para los estadios 3b y 4.

**Conclusiones:**

En pacientes con ERC es comun detectar bajos niveles de 25(OH)D, los cuales pueden contribuir a la generación de hiperparatiroidismo secundario.

## Introduction

Active vitamin D or calcitriol has important functions in patients with chronic kidney disease (CKD): it promotes the intestinal absorption of calcium and phosphorous; increases the distal tubular absorption of calcium in the kidney and exerts negative feedback on the parathyroid gland, lowering the synthesis and secretion of the parathyroid hormone (PTH). A sufficient supply of native vitamin D (25(OH) D) in the form of ergocalciferol (vitamin D2) or cholecalciferol (vitamin D3) is required for its synthesis in the kidney's proximal convoluted tubule 1-3. 

A progressive reduction in the levels of active vitamin D has been observed in patients with CKD proportional to the decrease of their glomerular filtration rate. It has been assumed that this happens due to a smaller amount of renal mass and due to the decrease in the number of proximal tubular cells that absorb the filtered native vitamin D (25(OH) D) to then be hydroxylated to its active form by the 1α-hydroxylase. 

However, a second explanation for the lower levels of active vitamin D could be the decrease of the seric levels of native vitamin D (25(OH) D) (known as a substrate deficit), which can be a result of a lower cutaneous synthesis of pre-vitamin D due to an increase of the skin's pigmentation and a smaller solar exposure. The medical prescription of low-protein diets (containing the native vitamin D) with the objective of reducing the input of phosphates is an additional cause for substrate deficit as well as the increase in the urinary loss of vitamin D-fixating proteins in patients with nephrotic syndrome 4-9. 

The purpose of this investigation was to determine if the substrate deficit (total native vitamin D) could be found in patients with CKD stages 2 to 5 not undergoing dialysis treatment and living in the city of Manizales, Caldas, Colombia, South America. 

Manizales is a city located in a tropical country; hence, it does not have seasons. It is located 2,200 meters (7,217 feet) above sea level, with an average temperature of 16,7° C (62° F), with coordinates: latitude 5°03´58" N y longitude 75°29´05" E. We suggest there is no correlation between geographical location and living in the heights with lower levels of vitamin D; instead, we suggest that habits such as living indoors, wearing extensive clothing, avoiding the sun and using sunscreen could have a significant correlation 10.

## Materials and Methods

### Study Area

The city of Manizales is located in the Cordillera Central (central Andes) of Colombia/South America 2,200 meters above sea level.

Patients who fulfilled the following characteristics were recruited for this study: 18 years of age or more, chronic kidney disease stages 2 to 5, not undergoing dialysis treatment, under treatment from the obligatory health plan (POS) in any of the following services: Internal Medicine and Nephrology in the University of Caldas, Renal Therapy Service (RTS), outpatient care in Children's Hospital (Hospital Infantil).

Exclusion criteria were: having been on vacation on a sunny area within the last 30 days, parathyroidectomy, hepatic disease, hospitalization within the last two months, treatment with either active or native vitamin D, fear of needles and/or refusing written consent.

All patients were explained the importance of quantifying the levels of 25-hydroxyvitamin D (25(OH) D) as well as the other variables and were asked to sign the written consent.

The collected demographic, anthropometric and clinical values were: sex, age, ethnicity, weight, size, body mass index, skin pigmentation, solar exposure, etiology for chronic kidney disease and, stage for chronic kidney disease.

Patients were interrogated and examined to confirm their degree of solar exposure in hands, face and arms. It was classified in three levels: level 1 (low), less than an hour per week; level 2 (moderate) between 1 and 3 hours per week and level 3 (adequate) more than 3 hours per week 11. Body mass index was obtained by applying the following formula: Body Mass Index = Weight (kg)/Height (m^2)^ and was defined as: thin (<18.49 kg); normal (18.50-24.99 kg); overweight (25.00-29.99 kg) and obese (>30.00 kg).

The collected laboratory tests were: creatinine, glomerular filtration rate (GFR) calculated by the MDRD formula 12, calcium, phosphorus, albumin, intact PTH, alkaline phosphatase, 24 hour urine protein, total levels of native vitamin D (25(OH) D) determined by electrochemiluminescence. 

Chronic kidney disease was defined according to the 2013 KDIGO guidelines 13. It was classified on stages according to the estimated GFR by the MDRD formula. The values, for stage and mL/min were: 2: 60-90, 3a: 45-59, 3b: 30-44, 4: 15-29 y 5: <15 mL/min.

Total levels of native vitamin D (25(OH) D) were defined according to the relationship between the seric levels of vitamin D, PTH and intestinal calcium transport according to what was referenced by the: International Osteoporosis Foundation and National Osteoporosis Foundation 2005 and 2010 14,15, American Geriatrics Society Consensus 16 and the National Osteoporosis Society 17. Values over 30 ng/mL were considered normal, insufficient between 10 and 30 ng/mL and deficient under 10 ng/mL. A PTH value over 70 pg/mL was considered compatible with secondary hyperparathyroidism. 

The medications the patients were receiving at the moment were annotated as well as those related with bone and mineral disorders in chronic kidney disease such as: chelators for oral phosphate and selective or non-selective agonist for the vitamin D receptor.

The bioethical committee from the University of Caldas, academic vice rectory and the RTS ethical and investigation committees for Colombia approved the project. 

### Study type

Prospective, analytical, cross-sectional study.

### Statistical analysis

The investigators used an excel database to digitize information; the statistical package SPSS ^®^ 15.0 was used for data processing and statistical tests calculations. Statistical descriptive methods were used to analyze data such as: measures of central tendency and quantitative variable dispersion. Absolute and relative frequencies were applied for the qualitative variables. A method of linear regression was used in order to determine correlations between the levels of vitamin D with each of the included laboratory variables; the variance analysis was used to determine the median comparisons between the levels of vitamin D and the stages of solar exposure and ethnicity.

Due to the fact that people with different stages of chronic kidney disease were included in the study and that laboratory results varied for each, affecting the statistical measurements such as median and standard deviation, we opted to calculate them for each stage.

##  Results

Three thirty three patients were evaluated, 158 men and 173 women with a median age of 71 ±14.4 years. A high variability was found in the anthropometrical characteristics of the chronic kidney disease patients, particularly age and weight. The average differences for each of these variables in each of the stages were statistically significant for all of them except size. A higher proportion of overweight patients were seen in stage 5 (47.8%) and 3b (43.1%) of CKD, but the differences were not statistically significant. Regarding ethnicity, most of the evaluated people were mestizos (71%), and from these the highest proportion was found in stage 4 of CKD (41.3%), with statistically significant differences between whites and mestizos (Table 1). The main etiologies for CKD were hypertensive nephropathy (33.2%), unknown (24.2%) and diabetic nephropathy (11.5%). Most of the patients were in stage 4 of CKD (125 (37.8%)), next in line were patients in stage 3b (123(37.2%)), 3a (48(14.5%)), 5 (23(6.9%)) and patients in stage 2 were last with (12(3.6%)) (Table 2).

**Table 1.** Description of the population according to demographic and anthropometric characteristics, depending of the chronic kidney disease stage (n=331).

Variables	2(n= 12)	3A(n= 48)	3B(n= 123)	4(n= 125)	5(n= 23)	F value	p value
	Mean (SD)	Mean (SD)	Mean (SD)	Mean (SD)	Mean (SD)		
Age	75 (12.36)	68.29 (15.84)	72.57 (13.58)	71.85 (13.94)	62.00 (15.57)	3.49	0.00826
Height	1.55 (0.08)	1.60 (0.10)	1.59 (0.09)	1.60 (0.10)	1.57 (0.08)	1.23	0.29890
Weight	52.11 (7.94)	60.51 (10.86)	65.16 (12.29)	64.40 (12.25)	64.91 (13.54)	4.23	0.00235
BMI	21.58 (2.75)	23.57 (3.37)	22.59 (4.05)	25.11 (3.94)	25.96 (3.76)	9.43	0.00000
Ethnic	No (%)	No (%)	No (%)	No (%)	No (%)	Chi2	p value
Mestizo	10 (4.2)	35 (14.9)	74 (31.5)	97 (41.3)	19 (81.0)	21.02	0.000314
White skinned people	2 (2.1)	13 (14.0)	48 (51.6)	26 (28.0)	4 (43.0)	25.52	0.000003
Black skinned people	0 (0.0)	0 (0.0)	1 (33.3)	2 (66.7)	0 (0.0		
Overweight	0 (0)	15 (31.2)	53 (43.1)	42 (33.6)	11 (47.8)	4.25	0.23540

**Table 2.** Level classification of vitamin D according to stage.

Stages	Deficit	Insufficient	Normal	Total
	(<10 ng/mL)	(10-30 ng/mL)	(>30 ng/mL)	
	No (%)	No (%)	No (%)	No (%)
2	0 (0.0)	8 (66.7)	4 (33.3)	12
3A	2 (4.2)	32 (66.7)	14 (29.2)	48
3B	9 (7.3)	87 (70.7)	27 (22.0)	123
4	11 (8.8)	90 (72.0)	24 (19.2)	125
5	7 (30.4)	15 (65.2)	1 (4.3)	23
Total	29 (8.8)	232 (70.1)	70 (21.1)	331
Chi2	11.89	0.84	2.01	
Valor p	0.0026	0.9333	0.3660	

The vitamin D concentration results showed that from the total of the analyzed patients only 21.1% was within normal values; 70.1% were insufficient and 8.8% were within deficit range. An increase in the percentage of patients within deficit range was found proportional to the deterioration of CKD stages from 2 to 5, with an initial value of 4.2%, to a final value of 30.4%, with statistically significant differences. This didn't happen in the group of patients within insufficient range where the percentage was relatively stable; the percentage of patients within normal range was reduced from 33.3% to 4.3% proportional to the decrease in their glomerular filtration rate; however the differences were not statistically significant for these two groups.

The F test was calculated in order to demonstrate statistically significant differences among the different stages for each laboratory test. These differences were found for all tests except calcium and albumin (Table 3).

**Table 3.** Descriptive measures of the laboratory results of the studied population, according to the chronic kidney disease stage.

	2	3A	3B	4	5	F	p
Laboratory test	Mean (SD)	Mean (SD)	Mean (SD)	Mean (SD)	Mean (SD)	test	value
Creatinine (mg/dL)	0.95 (0.19)	1.26 (0.16)	1.58 (0.25)	2.61 (0.61)	4.49 (0.67)	46.19	0.00000
Calcium (mg/dL)	9.38 (0.44)	9.27 (0.57)	9.46 (0.65)	9.32 (0.60	9.28 (0.66)	1.30	0.26970
Phosphate (mg/dL)	3.63 (0.57)	3.72 (0.65)	3.72 (0.68)	3.81 (0.66)	4.68 (0.75)	10.68	0.00000
Calcium x Phosphate	34.20 (6.65)	34.43 (6.07)	35.25 (6.99)	35.47 (6.57)	43.52 (7.61)	8.49	0.00000
Albumin (g/dL)	4.26 (0.36)	4.25 (0.48)	4.19 (0.40)	4.20 (0.43)	4.06 (0.67)	0.78	0.53642
PH (pg/mL)	45.91 (17.77)	62.13 (31.11)	77.43 (46.17)	116.5 (71.28)	215.35 (139.11)	30.21	0.00000
AP (mg/dL)	96.50 (41.85)	88.03 (35.84)	113.36 (51.81)	116.43 (61.89)	133.69 (46.72)	3.85	0.00452
24 hour urine protein (g)	0.33 (0.90)	0.32 (0.67)	0.23 (0.40)	0.87 (1.65)	2.20 (3.58)	10.87	0.00000
25(OH)D Levels (ng/mL)	26.71 (6.82)	26.54 (9.90)	24.21 (9.55)	23.07 (9.15)	17.34 (8.88)	4.33	0.00199
GFR (mL/min)	67.25 (7.50)	50.30 (4.00)	37.13 (4.18)	22.38 (4.53)	11.39 (1.78)	737.66	0.00000

A negative relationship was found between the levels of vitamin D and: creatinine, seric phosphorus, calcium x phosphorus product, alkaline phosphatase, PTH and 24 hour urine protein (*p* <0.05) (Table 4). Instead, a positive relationship was found between the levels of vitamin D, calcium and albumin. 

**Table 4.** The correlation between the laboratory results and Vitamin D levels. n=331.

Levels 25(OH)D	Creatinine	Calcium	Phosphate	Calcium x Phosphate	Albúmin	PTHi	Alkaline Phosphatase	24 hour urine protein
Correlation of Pearson	-0.164**	-0.014	-0.146**	-0.137*	0.009	-0.193**	-0.078	-0.214**
p value	0.003	0.795	0.008	0013	0.865	0.000	0.159	0.000

*The correlation is significant in 0.05 (bilateral)

**The correlation is significant in 0.01 (bilateral)

Even though the correlation between levels of vitamin D in each stage and the BMI was very weak; a negative correlation was found in stages 2, 3b, 4 and 5 of CKD; higher for stage 2 (Table 5).

**Table 5.** Correlation between Vitamin D levels and BMI, according to the chronic kidney disease stage.

Stages	Correlation	p value	n
2	-0.396	0.202	12
3A	0.110	0.456	48
3B	-0.157	0.082	123
4	-0.137	0.129	125
5	-0.110	0.618	23

Very low dependence and inversely proportional results between BMI and Vitamina D levels were detected

Concerning solar exposure, significant differences were observed between the averages of each vitamin D level (low, moderate and adequate) and solar exposure for stages 3b and 4 of CKD, for the other stages the differences between the averages were not statistically significant (Table 6).

**Table 6.** Comparison of averages of vitamin D levels, according to solar exposure.

Stages	Low (1)	Mild (2)	Appropriate (3)	F value	p value
	(n=204)	(n=59)	(n=68)		
2	24.5	-	29.3	0.971	0.415
3A	24.6	24.9	31.1	2.190	0.124
3B	21.0	27.5	34.6	2.476	0.000
4	20.7	23.2	29.4	9.764	0.000
5	14.5	22.0	21.5	2.039	0.156

There are statistically significant differences in the averages of vitamin D levels and the solar exhibition for the 3b and 4 stages

Regarding the ethnicity analysis, significant differences were found in the averages of vitamin D levels for whites and mestizos in stages 3a and 3b, but not for the rest of CKD stages. The averages were smaller for mestizos than whites, except for stage 3b, and for both races, as the stage increased, the vitamin D level averages decreased except in stage 3b for mestizos and 3a for whites (Table 7).

**Table 7.** Comparison of averages of vitamin D levels according to ethnic.

Stage	Mestizo	White skinned people	F value	p value
	(n=235)	(n=93)		
2	26.0	30.0	0.535	0.481
3A	24.2	32.8	8.165	0.006
3B	25.8	21.4	5.137	0.007
4	21.2	22.3	2.265	0.767
5	17.2	18.3	0.036	0.851

Significant differences are observed, between the averages of Vitamin D levels, of the mestizos and white skinned people races between 3a and 3b stages, what was not found to the rest of chronic kidney disease stages

## Discussion

Our study determined that in a group of 331 patients with CKD, as their glomerular filtration rate decreased from 90 mL/min to less than 15 mL/min, their levels of seric calcium, albumin and vitamin 25-(OH) D decreased proportionally. These patients were mainly mestizos and had normal weight (52.99%) and their etiology for CKD was more commonly hypertensive nephropathy. Additionally, calculating average or median, a progressive increase in seric phosphorus, calcium x phosphorus product, alkaline phosphatase and intact PTH was found.

Analyzing by sex, it was found that women showed higher average values of phosphorus and calcium x phosphorus product and lower average values for creatinine, 24 hour urine protein, iPTH, vitamin D and calcium compared to men along all ranges of GFR.

A statistically significative negative correlation was found between the levels of vitamin D and those of creatinine, phosphorus, calcium x phosphorus product, 24 hour urine protein and intact PTH. These findings demonstrate that as the CKD patients' renal function deteriorates, a series of events leads them to diminish their solar exposure or not take advantage of it properly due to increased skin pigmentation. Low protein diets, containing low amounts of vitamin D are often prescribed for this type of patients, a fact that probably contributes to lower seric levels of vitamin D. An additional factor could be proteinuria that could generate urinary loss of vitamin D binding protein. An elevation of PTH is a consequence of hyperphosphatemia, hypocalcaemia and low levels of the active form of vitamin D; levels that were not determined in this study 3. 

As expected, a positive correlation was found between the level of solar exposure and vitamin D 10. This indicates that in spite of higher skin pigmentation an adequate solar exposure (30 minutes three times a week) can stimulate the synthesis of vitamin 25(OH) D. Even though a negative correlation between obesity and levels of vitamin D has been described; we did not find it 10. In the analysis by ethnical group, mestizos representing 71% of patients, lower levels of vitamin D were found in comparison with the white population (28% of patients), demonstrating the importance of cutaneous pigmentation. Black population was only 0.91%, hence it could not be considered for statistical analysis.

Important limitations for this study were the high prevalence of mestizo's population and the lack of a control group living in tropical areas with a higher solar exposure. Even then we can presume that lifestyle related aspects have a high impact in the seric levels of vitamin D.

Vitamin D plays a very important role in mineral and bone metabolism, its active form, although synthesized mainly in the kidneys can also be originated in the prostate, breasts, immune system cells (macrophages), smooth muscle tissue cells, pancreatic B cells, gastrointestinal tract (colon) and skin thanks to the 1α-hydroxylase enzyme. Extrarenal production could give it other autocrine and paracrine actions such as promoting cellular proliferation and differentiation as well as regulating immune activity 2,18.

Secondary hyperparathyroidism is a common complication of chronic kidney disease. It results from the interaction of several different factors initiated by the loss of kidney tissue and the inability to excrete the daily load of phosphates, causing an increase in its seric levels. Hyperphosphatemia stimulates the posterior liberation of fibroblastic growth factor 23 (FGF23) by osteocytes, which inhibits proteins NaPiIIa and NaPiIIc in the proximal convoluted tubule generating phosphaturia. It also inhibits the activity of the 1α-hydroxylase renal enzyme, diminishing the synthesis of active vitamin D (1,25(OH)2D), leading to a reduction in the intestinal absorption of calcium and phosphorus, and seric levels of phosphorus. The final cost of lowering phosphorus is an increase in the levels of FGF23 and hypocalcaemia, this last one causing an increase in the synthesis and liberation of parathyroid hormone (PTH) 19,20.

The implications of the elevation of PTH are the emergence of bone complications such as renal osteodystrophy, vascular calcifications, cardiovascular disease and an increase in mortality 21,22. FGF23 also increases cardiovascular and global mortality for patients with CKD 23.

In an analysis from the Study for the Early Evaluation of chronic Kidney disease (SEEK) it is noted that the prevalence of secondary hyperparathyroidism (PTH over 65 pg/mL) starts to increase from CKD stage 3 and continues to increase all along the drop in the GFR, including practically all patients with a GFR below 20 mL/min 24. 

Insufficient seric levels of native vitamin D (D3 or D2), which are later, filtered in the glomeruli and received by the megalin receptors in the proximal convoluted tubule, can contribute significantly to lower levels of active vitamin D (1,25(OH)2D). Low substrate levels in patients with CKD can be a result of low solar exposure, increased skin pigmentation, low protein diets, and proteinuria 9,25; factors that often accompany CKD.

Therefore, it is very important to guarantee adequate seric concentrations of vitamin D and the substrate for 1,25(OH)2D to all patients with CKD, which have an important loss of proximal convoluted tubule cells due to their loss of nephron. In recent revisions the role of vitamin D in CKD has been analyzed finding more benefits than risks associated to its supplementation 26.

Vitamin D deficiency has also been implicated in bone abnormalities in various diseases including infections, diseases cardiovasculares 27-29, endothelial dysfunction 30, some types of neoplasms 31, insulin resistance 32,33, diabetic nephropathy 34, autoimmune diseases, depressives states 35 and decrease in mass muscular 36. Was also associated with high cardiovascular and all causes mortality 37,38.

The determination of seric levels of vitamin D in patients with CKD undergoing dialysis for their interpretation and subsequent supplementation has been a topic of controversy due to the different results obtained by different investigators 39,40.

The KDIGO guides, in relation to the topic of vitamin D in CKD suggest that seric levels of vitamin D should be determined in patients not undergoing dialysis; and if found insufficient should be corrected. There is no mention about using native vitamin D in patients undergoing dialysis 41.

A recent study demonstrated that the administration of cholecalciferol to patients with CKD stage 2 to 4 managed to restore seric levels of vitamin D and reduce those of PTH, demonstrating the benefit of its determination and posterior supplementation when necessary 42. Another study was made in patients with CKD stages 3 and 4 and with vitamin D values over 23 ng/mL and a stable GFR, they managed an adequate response to cholecalciferol in a course of 5 years follow up (considered for the suppression of PTH levels and to obtain vitamin D levels between 40 and 60 ng/mL) 43. An additional benefit from cholecalciferol in this population was the reduction in albuminuria even in patients with diabetic nephropathy 25,44; this is likely due to the intervention of pathways dependent or independent of blocking the renin-angiotensin-aldosterone system 45.

## Conclusion

Low levels of vitamin D are often found in patients with chronic kidney disease, these represent an important factor in the development of secondary hyperparathyroidism. Their identification and subsequent treatment with cholecalciferol (Vitamin D3) or ergocalciferol (Vitamin D2) in order to obtain adequate seric levels, allows a greater amount of substrate for the proximal convoluted tubule cells, and then inside improve the synthesis of the active form of vitamin D (calcitriol or 1,25(OH)2D) in patients with CKD without dialysis requirements, avoiding the development of all complications from hyperparathyroidism.

It is recommended for all patients with CKD in predialysis stages and with a GFR of less than 60 mL/min to evaluate their vitamin 25(OH)D levels and proceed to its formulation in order to avoid the consequences of its deficiency. 
