# Vitamin D (25(OH)D) in patients with chronic kidney disease stages 2-5

**DOI:** 10.25100/cm.v50i1.4444

**Published:** 2019-03-30

This corrects the article "**Vitamin D (25(OH)D) in patients with chronic kidney disease stages 2-5**" in volume 47(3) on page 163, Table 3.

The September 30, 2016, article by Restrepo and Aguirre (Colomb Med (Cali). 2016;47(3): 160-6, was published with errors in Table 3.

In body of Table 3, the sixth line of the first column entitled PH (pg/mL), this should read: PTHi (pg/mL).

The sixth line of the eight column reads: 11.65, this should read: 116.5. That date appears well in the English XML version, but in both the PDF ingles and the Spanish version appears the value 11.65, must be changed to the real value: 116.5.

