# Supplementary material for: Vitamin D (25(OH)D) in patients with chronic kidney disease stages 2-5
Source: Colomb Med (Cali). 2019 Mar 30;50(1):49. doi: 10.25100/cm.v50i1.4444 (PMC6536046; doi:10.25100/cm.v50i1.4444)
Supplement: Related texts

# Correction: Niveles de vitamina D (25(OH)D) en pacientes con enfermedad renal crónica estadios 2 a 5

En el artículo **Niveles de vitamina D (25(OH)D) en pacientes con enfermedad renal crónica estadios 2 a 5****,** volumen 47(3) en la pagina 163, Tabla 3 se debe hacer una corrección.

El 30 de Septiembre de 2016, en el artículo publicado por Restrepo y Aguirre (Colomb Med (Cali). 2016;47(3): 160-6, fue publicada la tabla 3 con errores

En el cuerpo de la Tabla 3, la sexta línea de la primera columna nombrada PH (pg/mL), se debe cambiar a: PTHi (pg/mL).

En la sexta línea de la octava columna se colocó: 11.65, esta cifra debe cambiarse a: 116.5. Esta cifra aparece bien en la versión ingles del XML pero no en los PDF versión inglés y español donde aparece el valor 11.65, Este valor debe ser cambiado por el valor real: 116.5.
